# A Comparison Between Normal and Reddened Mung Bean Soup: Beneficial Effects on the Regulation of Oxidative Stress, Inflammation, and Gut Microbiota in Mice with Heat Stress-Induced Damages

**DOI:** 10.3390/nu18142353

**Published:** 2026-07-17

**Authors:** Hao Ran, Mingyuan Zhang, Jiakai Wang, Zhitao Niu, Sumei Zhou, Dianzhi Hou

**Affiliations:** 1 Key Laboratory of Geriatric Nutrition and Health (Beijing Technology and Business University), Ministry of Education, Beijing 100048, China; 2 School of Food and Health, Beijing Technology and Business University, Beijing 100048, China

**Keywords:** heat stress, mung bean soup, phenolic analysis, oxidative stress, inflammation response, gut microbiota

## Abstract

Background: Mung bean soup (MBS), as a common form of mung bean consumption, is widely consumed in folk to relieve summer heat. However, MBS is prone to red discoloration during cooking and storage. Furthermore, how this color change affects its phenolic compounds composition and bioactivity remains poorly understood. Objectives: Herein, this study systematically compared the phenolic profiles and heat stress (HS)-alleviating effects of normal mung bean soup (NMBS) and reddened mung bean soup (RMBS). Methods: Male C57BL/6J mice (six-week-old) were randomly assigned to four groups: control (no HS), HS, HS-NMBS, and HS-RMBS. HS was induced at 40 °C for 2 h/day for 7 days, with NMBS or RMBS provided ad libitum throughout the experiment. Phenolic analysis, oxidative stress markers, inflammatory cytokines, and gut microbiota were evaluated. Results: The results showed that reddening significantly reduced the total phenolic and flavonoid contents by 18.28% and 16.77%, respectively, accompanied by the loss of key bioactive compounds (vitexin and isovitexin). NMBS and RMBS could effectively alleviate HS-induced physiological damages in mice to varying degrees, including reduced weight loss, improved hepatic and intestinal histopathology, and lower levels of oxidative stress and inflammation (*p* < 0.05). However, NMBS provided superior overall protection by downregulating the expression of HS-associated genes (PPAR-γ) and pro-inflammatory cytokines (IL-1β), while upregulating the anti-inflammatory cytokine IL-10. Furthermore, supplementation with NMBS or RMBS restored the gut microbiota dysbiosis induced by HS. Especially, NMBS was more effective in restoring microbial diversity, modulating the F/B ratio, and enriching beneficial bacteria including Lactobacillus, Dubosiella, and norank_f_Muribaculaceae. Conclusions: These results highlight the importance of avoiding the occurrence of redness in MBS to maximize its efficacy.

## 1. Introduction

Heat stress (HS) is a series of physiological, biochemical, and behavioral adaptive responses of organisms in a high-temperature environment due to an imbalance in thermoregulation. According to the World Health Organization (WHO) estimates, from 2000 to 2019, about 489,000 deaths per year globally are related to HS. HS can cause the body to produce excess reactive oxygen species, which directly damage mitochondria and promote the release of pro-inflammatory signaling molecules, ultimately leading to oxidative stress and inflammatory responses [1,2]. Furthermore, the intestine is one of the primary target organs affected by HS. Increased intestinal permeability often leads to harmful local and even systemic inflammatory responses, a phenomenon commonly referred to as “leaky gut” [3,4]. Several studies have indicated that HS could impair intestinal barrier function, thereby leading to increased intestinal permeability and local inflammation [5,6]. In addition, previous studies have reported that HS could induce gut microbiota dysbiosis, characterized by decreased diversity, significant structural changes, and abnormal elevation in the relative abundance of specific bacterial genera [7,8]. Therefore, the regulation of gut microbiota and the alleviation of inflammatory and oxidative stress is pivotal for improving HS. Currently, although the primary treatment methods, such as physical cooling and antibiotics, can mitigate HS, they fail to address the underlying pathological responses [9,10]. Recently, the enormous potential of plant-based components in preventing HS has become a safer and more effective intervention strategy that deserves further exploration.

Mung bean (*Vigna radiata* L.), as a traditional medicinal and edible plant, is widely consumed in Asia, particularly in China. Mung bean is rich in diverse phenolic compounds, including phenolic acids (such as chlorogenic, *p*-coumaric, and ferulic acids), flavonoids (vitexin and isovitexin), and tannins [11,12]. Mung bean soup (MBS), as one of the main edible forms of mung bean, has always been widely used by the folk to clear away summer heat. Previously, the ability of MBS to alleviate HS was simply attributed to its antioxidant properties, such as enhancing total antioxidant capacity (T-AOC) and glutathione (GSH) levels, while reducing malondialdehyde (MDA) content [13,14], but its specific mechanism of action remains unknown. Notably, the health benefits of MBS may extend beyond its direct antioxidant effects to include modulation of the gut microbiota. In previous studies, Hou, et al. [15] demonstrated that mung bean coat significantly altered the fecal microbiome and metabolome in mice with diet-induced non-alcoholic fatty liver disease, thereby increasing the abundance of beneficial bacteria such as *Akkermansia* and improving hepatic lipid metabolism. Similarly, it has been reported that polysaccharides extracted from mung bean significantly promoted the growth of beneficial bacteria in Balb/c mice and improved the intestinal microenvironment through the production of short-chain fatty acids (SCFAs) [16]. These findings collectively suggested that mung bean constituents could interact with the gut microbial ecosystem, thereby influencing host metabolic. However, whether MBS, as the most consumed form of mung bean, exerted similar gut microbiota-modulating effects, particularly under heat stress conditions, remained to be elucidated. In addition, MBS is prone to discoloration during cooking and storage, which results in a reddish color. This color change signifies not merely a degradation of visual quality, but also indicates an underlying transformation of bioactive components, which may consequently lead to alterations in its health benefits. For instance, one study demonstrated that the color of wild Andean blueberry wine shifted from red/violet to red/orange during bottle storage, with a concomitant enhancement in its antioxidant capacity [17]. However, in another study, wine with elevated copper residues was associated with marked fading of color, a reduction in red and yellow hues, and a corresponding substantial decline in antioxidant activity [18]. This color change may be closely linked to alterations in the composition and content of phenolic compounds [19,20]. Currently, the phenolic composition underlying the distinct color difference between reddened mung bean soup (RMBS) and normal mung bean soup (NMBS) remains poorly characterized. Furthermore, the differential beneficial effects and mechanisms of RMBS and NMBS in alleviating HS in mice also need to be revealed.

We hypothesized that the red discoloration of MBS during storage led to a significant loss of phenolic compounds, thereby compromising its efficacy in alleviating HS. Furthermore, we postulated that NMBS and RMBS exerted differential protective effects against HS through distinct mechanisms involving the regulation of oxidative stress, inflammatory responses, and gut microbiota modulation. To test these hypotheses, we systematically compared the phenolic profiles of NMBS and RMBS and evaluated their protective effects against HS in a mouse model, focusing on oxidative stress, inflammation, and gut health. The results can provide a scientific basis for the further development of MBS as a functional anti-HS beverage.

## 2. Materials and Methods

### 2.1. Materials and Reagents

Mung beans (Zhonglv 12, Tangshan, China) were provided by Institute of Crop Sciences, Chinese Academy of Agricultural Sciences (Beijing, China). Test kits for Catalase (CAT), malondialdehyde (MDA) and superoxide dismutase (SOD) were supplied by Nanjing Jiancheng Bioengineering Institute (Nanjing, China). Test kit for total antioxidant capacity (T-AOC) was purchased from Beijing Solarbio Science and Technology Co., Ltd. (Beijing, China). Kits for alanine aminotransferase (ALT) and aspartate aminotransferase (AST) were purchased from Shenzhen Myriad Bio-Medical Electronics Co., Ltd. (Shenzhen, China). Kit for riboSCRIPT Reverse Transcription (500T), Oligo (dT) 18 Primer (25 μM, 20 μL) and Random Primer (200 μM, 20 μL) were obtained from Guangzhou Ruibo Biotechnology Co., Ltd. (Guangzhou, China). The FastPure Stool DNA Isolation Kit was procured from MJYH (Shanghai, China), and FastPfu DNA Polymerase was obtained from Beijing TransGen Biotech Co., Ltd. (Beijing, China).

### 2.2. Preparation of NMBS and RMBS

The preparation of *NMBS and RMBS* was referred to the method of Li, Cao, Yi, Cao and Jiang [13] with appropriate modifications. A total of 12.5 g of intact mung beans was cleaned and subjected to boiling in 100 mL of deionized water for 35 min. The cooked MBS was finally filtered through a 100-mesh strainer. The filtrate was cooled, adjusted to a final volume of 100 mL, and then centrifuged (6000 r/min) for 10 min to obtain the MBS. The supernatant was taken as NMBS. The supernatant was sealed in aluminum cans. These sealed cans were then autoclaved in water at 100 °C for 30 min. Finally, they were stored at 37 °C for 7 days to obtain RMBS.

### 2.3. Determination of Total Phenolic Content (TPC) and Total Flavonoid Content (TFC)

The TPC was determined based on the previous study with minor modifications [21]. A mixture was prepared by combining 125 μL of MBS supernatant with 500 μL of distilled water and 125 μL of Folin–Ciocalteu reagent, followed by vortexing. After 6 min, 1.25 mL of sodium carbonate (7%) was added. Subsequently, distilled water was added to bring the final volume to 3.0 mL, and the mixture was vortex-mixed. After incubation for 90 min at room temperature in the dark, the absorbance at 760 nm was measured by microplate reader. Gallic acid was used as the standard, and the TPC was expressed as gallic acid equivalent (mg GAE/ 100mL).

The TFC was determined according to the study of Gomes, et al. [22] with slight modifications. A total of 100 μL of MBS supernatant was mixed with 30 μL of 5% sodium nitrite solution. Then, 30 μL of 10% aluminum chloride was added. After 6 min, 400 μL of 4% sodium hydroxide solution was added and reacted for 20 min, and finally the absorbance was measured at 510 nm. Rutin was used as the standard, and the TFC was expressed as rutin equivalent (mg RE/100 mL).

### 2.4. Identification and Quantification of Phenolic Compounds by LC-MS/MS

The phenolic compounds were identified in accordance with the method reported by Xia, et al. [23]. Prior to liquid chromatographic analysis, the supernatant of NMBS and RMBS was filtered through a 0.22 μm PTFE syringe filter and subsequently used for chromatographic detection. The chromatographic column employed was an Agilent Eclipse Plus C18 1.8 μm (2.1 × 100 mm). The mobile phase consisted of 0.1% formic acid in water (solvent A) and acetonitrile (solvent B). Gradient elution was performed as follows: at 0 min, 5% B and 95% A; at 20 min, 100% B and 0% A. The column was then equilibrated for 3 min between samples as follows: at 20.01 min, 5% B and 95% A; at 23 min, 5% B and 95% A. The column oven temperature was maintained at 40 °C, with a flow rate of 0.3 mL/min and an injection volume of 5 μL. Ultraviolet detection was carried out at a wavelength of 280 nm. A calibration curve was constructed with mass concentration (ng/mL) as the abscissa and peak area as the ordinate. The final results were expressed as mg/100 g.

### 2.5. Animals Experimental Design

Thirty-two male C57BL/6J mice (6 weeks old, body weight 20 ± 2 g) were purchased from Beijing Vital River Laboratory Animal Technology Co., Ltd. (Beijing, China). The mice were housed under standard conditions with a temperature of 22 ± 2 °C, relative humidity of 60 ± 5%, and a 12 h light/12 h dark photoperiod, and were provided with a standard diet and water. After one week of acclimatization feeding, 32 mice were randomly divided into the following 4 groups (*n* = 8): control group (NCD), heat stress group (HS), heat-stressed mice supplemented with normal mung bean soup (HS-NMBS), and heat-stressed mice supplemented with reddened mung bean soup group (HS-RMBS).

**Figure 1:**
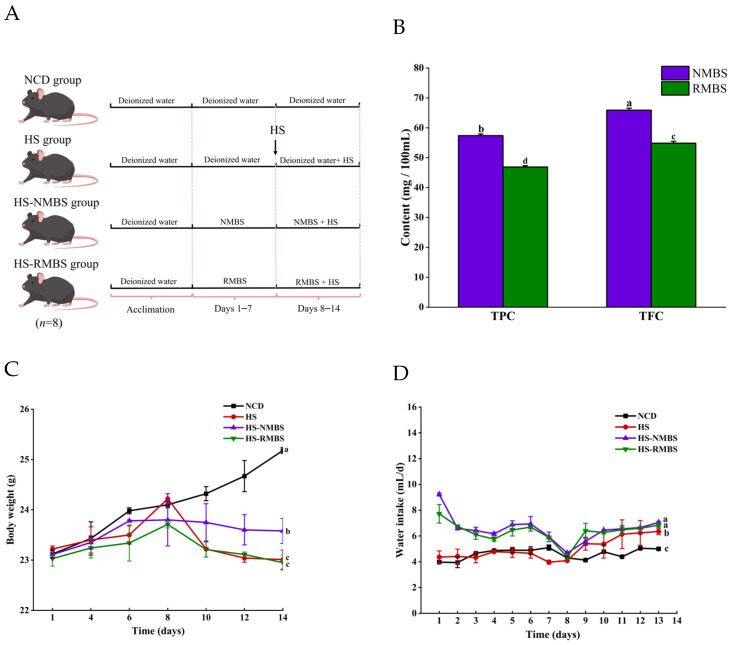
Effects of NMBS and RMBS on body weight and water intake of mice. (**A**) Specific experimental protocol. (**B**) The total phenolic content (TPC) and total flavonoid content (TFC) of NMBS and RMBS (mg/100 mL). (**C**) Changes in body weight. (**D**) Water intake. Different letters indicated statistically significant differences between groups (*p* < 0.05), whereas values sharing the same letter were not significantly different (*p* ≥ 0.05).

The experimental scheme is shown in Figure 1A, adapted from Huang, et al. [24], with minor modifications. On days 1–7, the NCD and HS groups were free to drink deionized water, while the HS-NMBS and HS-RMBS groups were free to drink NMBS and RMBS, respectively. All groups were free to perform ingestion activities. On days 8–14, all groups except the NCD group underwent heat stress (temperature: 40 ± 1 °C; humidity: 60 ± 5%) for 2 h from 10 to 12 a.m. for 7 consecutive days [25]. Once the heat stress ended, they were immediately released back to the room-temperature environment and fed and watered freely. At the end of the experiment, all mice were fasted for 6 h. Subsequently, mice were anesthetized with sodium pentobarbital, blood was collected from the retro-orbital sinus, and the animals were euthanized by cervical dislocation. Serum was separated for analysis. Liver, jejunum, and colon were taken for morphometric analysis, and the rest was snap-frozen in liquid nitrogen and stored at −80 °C for subsequent analysis.

All animal experiments were conducted in accordance with the National Research Council Guidelines and were approved by the Institutional Animal Care and Use Committee of Beijing Technology and Business University (Protocol No. 2025-84).

### 2.6. Measurement of Body Weight and Water Intake

At the end of the acclimatization feeding period, all groups were weighed for the first time. Body weight measurements were taken every other day onwards. Starting from day 1, the MBS was changed daily at 8 a.m. and 8 p.m. for each group of subjects and the amount consumed was recorded accurately.

### 2.7. Measurement of Organ Index

The livers, kidneys, thymus, and spleens of the mice were carefully washed with saline to thoroughly remove blood stains from the organ surfaces. After cleaning, the residual water on the surface of these organs was removed by blotting with filter paper, and they were subsequently weighed to record the wet weight data.

Organ index (%) = organ wet weight (g)/mouse body weight (g) × 100%.

### 2.8. Measurements of Serum AST, ALT, and Antioxidant Index

Mice serum AST and ALT were detected using a BS-1000M (Shenzhen Myriad Bio-Medical Electronics Co., Ltd., Shenzhen, China) automatic biochemical analyzer. The activities of SOD, CAT, MDA, and T-AOC in serum were measured using assay kits according to the manufacturer’s instructions.

### 2.9. Histopathological Examination

Liver, jejunum, and colon tissues of mice were taken and fixed with 4% paraformaldehyde, and then embedded after being dehydrated and dipped in wax. The embedded tissues were cut into 5 μm slices in a slicer. The paraffin sections were sequentially dewaxed and rehydrated, and then stained with hematoxylin and eosin (H and E), respectively. After staining, the sections were dehydrated and sealed. Photographs were taken under the microscope to observe the pathological changes.

### 2.10. Measurement of Cytokine Gene Expression

The total RNA was extracted from liver tissue samples using the Trizol reagent, and its concentration was assessed with a microvolume UV spectrophotometer (NanoDrop One, Thermo Fisher Scientific Co., Ltd., Shanghai, China). Subsequently, complementary DNA (cDNA) was synthesized from the total RNA using the riboSCRIPT Reverse Transcription Kit (Ribbio Biotechnology Co., Ltd., Guangzhou, China). Gene-specific primers for quantitative real-time PCR (qPCR) were designed using the NCBI-primer tool, based on the gene sequences obtained from the NCBI database. The qPCR primer sequences are shown in Table S1. The expression levels of target genes were determined by qPCR. The qPCR amplification protocol was carried out under the following conditions: initial denaturation at 95 °C for 10 min, followed by 40 cycles of denaturation at 95 °C for 5 s, annealing at 60 °C for 30 s, and extension at 72 °C for 30 s. All reactions were performed in triplicate, and the relative gene expression was calculated using the 2^−ΔΔCt^ method.

### 2.11. Gut Microbiota Analysis

Fecal samples were collected from individually housed mice in metabolic cages to prevent urine contamination. The fresh feces were then transferred into pre-chilled sterile centrifuge tubes, snap-frozen in liquid nitrogen, and stored at −80 °C until subsequent analysis. DNA was extracted from the fecal samples using the FastPure Stool DNA Isolation Kit (Shanghai Major Bio-Pharmaceutical Technology Co., Ltd., Shanghai, China) according to the manufacturer’s instructions. The integrity of the extracted DNA was verified by 1% agarose gel electrophoresis, and its concentration and purity were subsequently determined.

The V3-V4 region of the 16S rRNA gene was amplified via PCR using the upstream primer 338F (5′-ACTCCTACGGGAGGCAGCAG-3′) and the downstream primer 806R (5′-GGACTACHVGGGTWTCTAAT-3′), both of which were tagged with a barcode sequence. The amplification procedure referred to the method described previously [15]. The PCR products were recovered using agarose gel (2%) electrophoresis and subsequently purified with a DNA gel extraction and purification kit (PCR Clean-Up Kit, Shanghai Major Bio-Pharmaceutical Technology Co., Ltd., Shanghai, China). The purified products were then quantified using a Qubit 4.0 fluorometer (Thermo Fisher Scientific, Waltham, MA, USA). The purified amplicons were pooled in equal amounts and paired-end sequenced (2 × 250 bp) on an Illumina NextSeq 2000 platform following the standard protocols at Shanghai Majorbio Bio-Pharm Technology Co., Ltd. (Shanghai, China). The raw sequence reads generated in this study have been deposited in the NCBI Sequence Read Archive (SRA) under BioProject accession number PRJNA1491072.

The average number of raw reads per sample was 58,837, and the average number of filtered reads per sample was 53,679. Raw paired-end reads were quality-filtered using fastp software (version 0.19.6, https://github.com/OpenGene/fastp, accessed on 16 April 2026) [26] with default parameters, and clean reads were subsequently assembled using FLASH software (version 1.2.11, http://www.cbcb.umd.edu/software/flash, accessed on 16 April 2026) [27]. Denoising and chimera removal were performed using the DADA2 plugin (version 2024) within the QIIME 2 platform (version 2024, https://qiime2.org, accessed on 16 April 2026) [28] with default parameters, generating amplicon sequence variants (ASVs). Taxonomic assignment of ASVs was conducted using the SILVA database (release 138, https://www.arb-silva.de/, accessed on 16 April 2026) [29] as the reference, with a confidence threshold of 0.7. Alpha diversity indices were calculated using the mothur software (version 1.30.2) [30], and intergroup differences were assessed using the Wilcoxon rank-sum test. Beta diversity was evaluated by principal coordinate analysis (PCoA) based on Bray–Curtis distances, and the significance of community structure differences among groups was tested using Permutational Multivariate Analysis of Variance (PERMANOVA), implemented in the vegan package of R software (version 3.3.1). Linear Discriminant Analysis Effect Size (LEfSe, http://galaxy.biobakery.org/, accessed on 16 April 2026) [31,32] was employed to identify bacterial taxa with significantly differential abundances from phylum to genus level, with a logarithmic LDA score threshold of > 2.0 and a significance level of *p* < 0.05.

### 2.12. Statistical Analysis

All data represented the mean values of triplicate experiments, and the results were shown as the mean ± standard deviation. One-way analysis of variance (ANOVA) and Duncan’s multiple range test were performed using SPSS statistical software (version 27.0, IBM Corporation, Chicago, IL, USA) to determine the statistical significance of differences among the indices (*p* < 0.05).

## 3. Results and Discussion

### 3.1. TPC and TFC of NMBS and RMBS

As indicated in Figure 1B, compared with NMBS, the TPC and TFC of RMBS all significantly decreased (*p* < 0.05) due to the occurrence of redness. Specifically, a reduction of 18.27% (from 57.40 to 46.91 mg GAE/100 mL) and 16.77% (from 65.91 to 54.86 mg RE/100 mL) was observed in the TPC and TFC, respectively. This result clearly demonstrated that the reddening phenomenon in MBS was accompanied by a significant loss of phenolic and flavonoid compounds. This might be closely related to the oxidation of phenolic compounds. During processing and storage, the phenolic compounds in MBS were readily oxidized into quinones, thereby leading to a notable decline in their content, due to the accelerated non-enzymatic and enzymatic oxidation [33,34]. Concurrently, the increase in oxidation products could cause the color of the MBS to darken. The observed reductions in both TPC and TFC in this study suggested that the bioactivity of MBS in alleviating heat stress might be consequently compromised.

### 3.2. Identification and Quantification of Phenolic Compounds in MBS

A total of 17 monomeric phenolics, including six phenolic acids, two catechins, and nine flavonoids, were detected in NMBS and RMBS (Table 1). Compared with NMBS, the contents of all six phenolic acids were significantly reduced in RMBS (*p* < 0.05). Among them, chlorogenic acid suffered the largest absolute decrease, dropping from 1.07 to 0.54 mg/100 g (a reduction of 49.23%), followed by *p*-coumaric (89.16%), benzoic (84.11%), vanillic (78.38%), ferulic (55.77%), and gallic acids (51.49%). Compared with NMBS, the same trend was observed among catechin compounds in RMBS, with catechin and epicatechin decreasing markedly by 75.29% and 27.93%, respectively. For the flavonoids, the C-glycosyl flavones vitexin and isovitexin, which were the two most abundant monomeric phenolic compounds in both NMBS and RMBS. During the process of turning red, their contents were significantly reduced from 37.71 mg/100 g to 15.23 mg/100 g and from 47.85 mg/100 g to 17.08 mg/100 g (*p* < 0.05), respectively. In addition, the levels of rutin and naringin decreased by 52.59% and 37.33%, respectively, whereas the contents quercetin, and diosmetin only showed slight increases. Vitexin and isovitexin were well-documented for their potent antioxidant and anti-inflammatory activities, including scavenging reactive oxygen species, and reducing pro-inflammatory cytokine production [35,36]. The reductions in the content of various phenolic compounds caused by reddening may weaken the efficacy of MBS in alleviating heat stress.

### 3.3. The Effect of NMBS and RMBS on Body Weight and Water Intake

As shown in Figure 1C, from day 1 to 7, with conditions of free feeding and no external stimuli, all groups exhibited a gradual weight gain. After day 7, although the body weight of NCD group continued to increase, the other three heat-stressed groups showed significant weight loss. Notably, following the cessation of heat stress, the HS-NMBS group maintained a significantly higher body weight than that of HS-RMBS group (*p* < 0.05), which itself was not significantly different from the HS group.

During days 1 to 7, the consumption of MBS by mice was significantly higher than that of plain water, which may be attributed to the appealing aroma and mild sweetness of the soup. On the 8th day when heat stress treatment commenced, water intake sharply decreased in the three groups of mice subjected to heat stress. This phenomenon might be attributed to the inability of mice to acclimate during their initial exposure to elevated temperatures, resulting in symptoms similar to heat stroke. As heat stress treatment continued, water intake gradually increased, with the HS-NMBS and HS-RMBS groups showing significantly higher intake than the HS group (Figure 1D). However, there was no significant difference between the HS-NMBS and HS-RMBS groups in their final water consumption levels.

Our results indicated that MBS might alleviate the weight loss caused by heat stress. However, body weight was a non-specific indicator of animal health, influenced by multiple factors including food intake, water consumption, hydration status, and metabolic adaptations. Therefore, the lack of food intake data collection limited a more accurate explanation of the improvement of body weight by MBS. Furthermore, the animals exhibited a phenotypic response to heat stress characterized by increased water intake, which served to meet the elevated demand for evaporative heat dissipation [37]. However, no significant difference in final fluid intake was observed between the HS-NMBS and HS-RMBS groups, indicating that the efficacy discrepancy between these two groups might not be attributed to the volume of beverage consumed. Therefore, the differential effects of NMBS and RMBS are more likely related to their distinct chemical compositions and relative contents of bioactive components, rather than to the amount of intake alone.

### 3.4. The Effect of NMBS and RMBS on Organ Indices

Organ indices are commonly used as indicators of physiological status. According to Table 2, all the organ indices of mice in the HS group were significantly reduced as compared to the NCD group (*p* < 0.05), indicating that heat stress caused damage to the organs of the mice. Interestingly, except for the kidney index, the liver index, thymus index, and spleen index recovered after intervention with MBS in heat-stressed mice (*p* < 0.05), but there was no significant difference between the HS-NMBS and HS-RMBS groups.

An increase in thymus and spleen indices may reflect an enhancement of cellular immune function. The study results of Hu, et al. [38] demonstrated that heat-stressed mice exhibited reductions in thymic, splenic, and hepatic indices, all of which showed recovery following L-theanine treatment. Similarly, in this study, the thymus, spleen, and liver indices of mice were reduced following heat stress exposure, but these indices rebounded after treatment with MBS. These findings suggested that MBS may exert a modulatory effect on the function of immune organs, thereby offering a certain degree of amelioration for the immune dysfunction in mice caused by heat stress. In addition, although the liver is not a classical immune organ, its index recovery further indicated that MBS attenuated multi-organ damage induced by heat stress.

### 3.5. The Effect of NMBS and RMBS on Liver Damage

**Figure 2:**
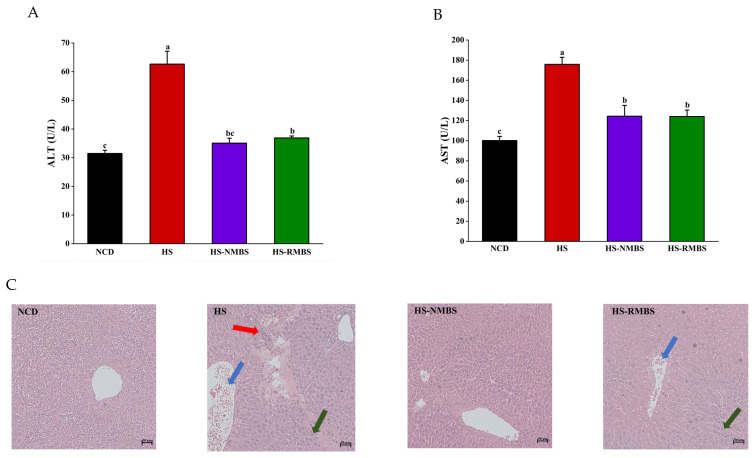
Effects of NMBS and RMBS on hepatic tissue. (**A**) Serum alanine aminotransferase (ALT) levels. (**B**) Serum aspartate aminotransferase (AST) levels. (**C**) Representative photomicrographs of liver sections (H and E staining, scale bar = 50 μm). Note: Blue arrows indicated bleeding in central veins and hepatic sinusoids; red arrows indicated inflammatory cell infiltration; green arrows indicated hepatocyte swelling. Different letters indicated statistically significant differences between groups (*p* < 0.05), whereas values sharing the same letter were not significantly different (*p* ≥ 0.05).

As shown in the Figure 2A,B, heat stress treatment significantly elevated the serum ALT and AST levels in mice when compared with the NCD group (*p* < 0.05). The increased ALT and AST enzyme activities by heat stress were significantly decreased in the HS-NMBS and HS-RMBS groups (*p* < 0.05), but there was no significant difference between the two treatment groups. Figure 2C showed representative photomicrographs of H and E-stained hepatic tissue sections. The hepatocytes in the NCD group exhibited a relatively orderly arrangement and overall normal architecture, with no evident inflammatory-cell infiltration or other abnormalities. Consistent with the levels of these serum biomarkers, the HS group exhibited markedly disorganized hepatic cords, including degenerative changes and swelling of hepatocytes, focal vascular congestion with erythrocyte extravasation, and substantial inflammatory-cell infiltration. In contrast, the HS-NMBS group showed closely packed, regularly arranged hepatocytes with intact intercellular structures, and no overt morphological anomalies, indicating minimal hepatocellular damage. The HS-RMBS group presented slight widening of intercellular spaces, mild erythrocyte leakage in a few sinusoids, and moderate cellular swelling with altered morphology, yet the extent of injury was appreciably less severe than in the HS group.

ALT and AST levels were commonly utilized as biomarkers for the assessment of hepatic injury induced by heat stress [39]. Elevated serum concentrations of ALT and AST were indicative of increased cellular damage. In this study, heat stress was found to elevate the levels of ALT and AST in mice, which was consistent with the conclusions drawn by Wang, et al. [40]. Liver function was alleviated by MBS, as indicated by the reduced serum levels of ALT and AST. Furthermore, MBS significantly attenuated heat stress-induced histopathological lesions in the liver, with NMBS exhibiting superior efficacy than RMBS. Our findings showed that MBS supplementation attenuated heat stress-induced changes in liver.

### 3.6. The Effect of NMBS and RMBS on Serum Antioxidant Capacity

**Figure 3:**
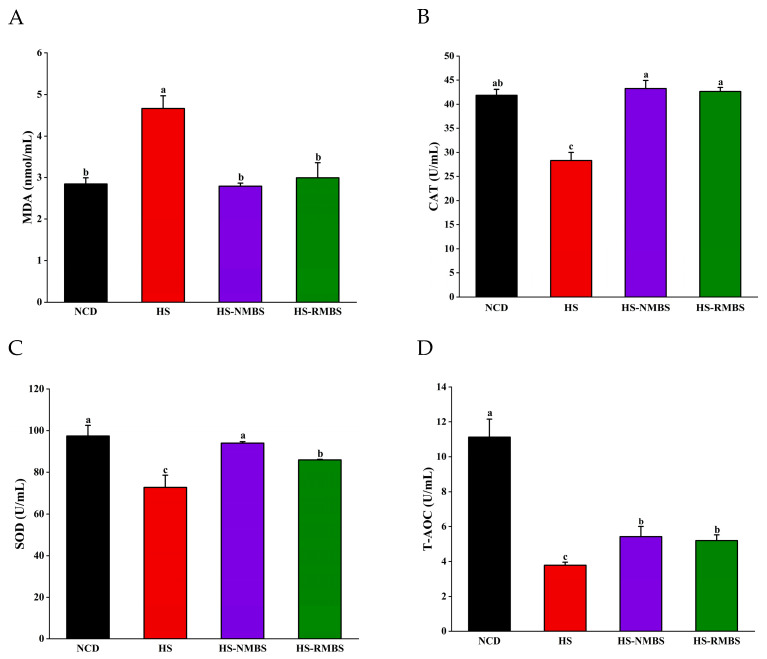
Effects of NMBS and RMBS on serum antioxidant capacity. (**A**) Serum malondialdehyde (MDA) level (nmol/mL). (**B**) Serum catalase (CAT) activity (U/mL). (**C**) Serum superoxide dismutase (SOD) activity (U/mL). (**D**) Serum total antioxidant capacity (T-AOC) (U/mL). Different letters indicated statistically significant differences between groups (*p* < 0.05), whereas values sharing the same letter were not significantly different (*p* ≥ 0.05).

Oxidative damage is one of the most common adverse physiological states induced by heat stress [1]. To further investigate the effect of MBS on heat stress-induced oxidative stress, we examined oxidative indicators in serum. As shown in Figure 3, MDA levels were significantly increased while SOD, CAT, and T-AOC levels were significantly decreased in the HS group mice when compared with the NCD group (*p* < 0.05). After treatment with NMBS and RMBS, the mice showed a significant decrease in MDA levels, while SOD, CAT, and T-AOC levels were increased (*p* < 0.05). A significant difference was observed specifically in the improvement of SOD levels between NMBS and RMBS (*p* < 0.05), whereas no marked differences were found in the mitigation of other oxidative stress indices.

A previous study showed that heat stress in broilers was associated with increased serum MDA levels and concomitant decreases in the activities of SOD and CAT [41]. Similar findings have been reported in exotic rabbit breeds during the peak heat stress period in Nigeria [42]. Our results suggested that MBS had a significant mitigating effect on heat stress-induced oxidative stress damage by enhancing antioxidant enzyme activities and boosting total antioxidant capacity. Furthermore, NMBS demonstrated superior efficacy compared to RMBS in mitigating heat stress-related damage.

### 3.7. The Effect of NMBS and RMBS on Jejunum and Colon

**Figure 4:**
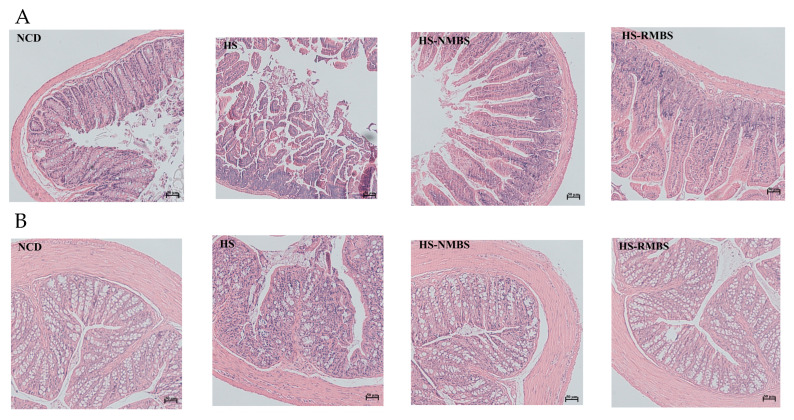
Effects of NMBS and RMBS on jejunum (**A**) and colon (**B**) tissues (scale bar = 50 μm).

The gut is recognized as highly vulnerable to environmental factors like heat stress, with intestinal injury representing a prevalent complication during thermal challenge [43,44]. As illustrated in Figure 4A, the NCD group exhibited well-preserved mucosal architecture characterized by regularly arranged villous epithelial cells and absence of necrosis or exfoliation. However, the HS group displayed severe jejunal damage, including villous shortening and fracture, epithelial cell swelling and shedding (particularly at the villous tips), and mild inflammatory infiltration in the lamina propria, which were consistent with the findings by Chauhan, Rashamol, Bagath, Sejian and Dunshea [1]. The HS-NMBS group showed relatively intact jejunal morphology with only focal degeneration, slight epithelial discontinuity, and minimal inflammatory cell infiltration. The HS-RMBS group presented moderate villous disarray, with epithelial degeneration, necrosis, and exfoliation being more evident than in the HS-NMBS group but less severe than in the HS group, accompanied by discernible inflammatory infiltration. In the colon (Figure 4B), the NCD group exhibited structurally intact and uniformly arranged colonic glands without inflammatory cell accumulation. The HS group, however, showed marked epithelial desquamation, glandular degeneration and fragmentation, and pronounced inflammatory infiltration. Both MBS-treated groups exhibited significant improvement in colonic histology, including restored glandular morphology and reduced inflammatory infiltrates, with the HS-NMBS group demonstrating the most pronounced protective effect.

Intestinal morphology is a key parameter in the assessment of gut health [45]. Under high-temperature conditions, the intestine acts as a central organ in the stress response and is highly susceptible to damage [46]. Previous study has demonstrated that heat stress impaired intestinal morphology in mice, characterized by villous stromal edema, focal necrosis, partial epithelial detachment, along with evident edema and hyperemia [47]. Our research supported this finding. Moreover, our findings indicated that MBS, particularly NMBS, effectively attenuated heat stress-induced intestinal injury in both the jejunum and colon. The barrier function–related effects observed in the present study warrant further validation in future studies.

### 3.8. The Effect of NMBS and RMBS on mRNA Expression of Cytokine, HSP70, and PPAR-γ

**Figure 5:**
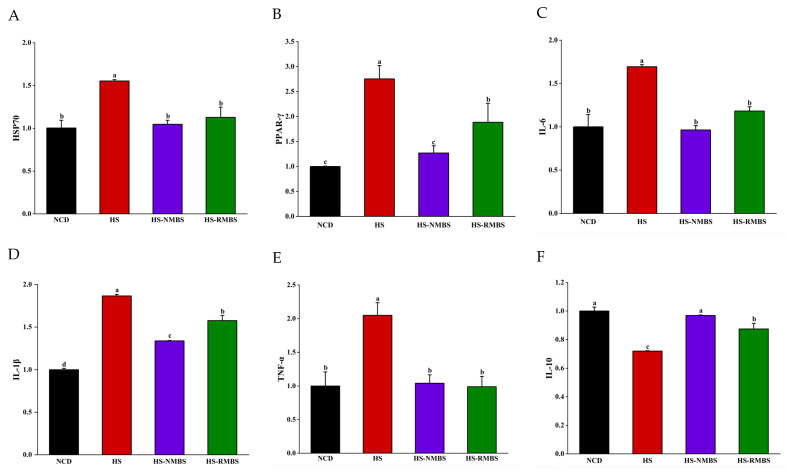
Effects of NMBS and RMBS on key genes expression. Relative mRNA expression levels of (**A**) heat shock protein 70 (HSP70), (**B**) peroxisome proliferator-activated receptor-γ (PPAR-γ), (**C**) interleukin-6 (IL-6), (**D**) interleukin-1β (IL-1β), (**E**) tumor necrosis factor-α (TNF-α), and (**F**) interleukin-10 (IL-10) in liver tissues. Gene expression was normalized to the internal control and calculated using the 2^−ΔΔCt^ method. Different letters indicated statistically significant differences between groups (*p* < 0.05), whereas values sharing the same letter were not significantly different (*p* ≥ 0.05).

The expression level of heat shock proteins (HSPs) is often regarded as a key basis for assessing the body’s ability to resist injury [48,49]. Among them, HSP70 has been mainly studied for its relevance to heat stress and environmental stresses [50,51]. The upregulation of HSP70 expression under heat stress represented a conserved cellular protective mechanism, as extensively documented in various species [52]. The study by Parida, et al. [53] revealed that heat stress increased the HSP70 expression in goats, showing a gradual enhancement in gene expression levels in a duration-dependent manner. Similar results have also been observed in broilers [54]. Similarly, the protective role of PPAR-γ activation in attenuating inflammation and oxidative stress has been well established [55]. Heat stress marker analysis revealed that heat stress exposure significantly elevated the expression of both HSP70 and PPAR-γ in the HS group relative to the NCD group (*p* < 0.05; Figure 5A,B). MBS intervention effectively reduced the increased expression caused by heat stress, with both treatment groups showing significant downregulation of HSP70 and PPAR-γ (*p* < 0.05). Furthermore, although there was no significant difference in HSP70 expression between the HS-NMBS and HS-RMBS groups, the HS-NMBS group exhibited a more pronounced reduction in PPAR-γ expression than the HS-RMBS group. In this study, MBS treatment significantly downregulated the expression levels of HSP70, suggesting that MBS may mitigate cellular stress, thereby reducing the need for intensive molecular chaperone activity. In addition, the upregulation of PPAR-γ expression under heat stress conditions might represent a metabolic adaptive mechanism aimed at enhancing lipid storage capacity and sustaining energy homeostasis during periods of elevated energy expenditure [38]. Accordingly, the HS-NMBS and HS-RMBS groups, in which HS-induced damage was alleviated by MBS intervention, exhibited a less pronounced increase in PPAR-γ expression compared with the HS group. The elevation of PPAR-γ under heat stress and its normalization after MBS intervention indicated that the metabolic burden imposed by heat stress was effectively alleviated. Previous studies have demonstrated that heat stress upregulated the PPAR-γ gene expression in both mice [38] and neonatal piglets [56], which were consistent with our results. The ability of MBS to improve both HSP70 and PPAR-γ overexpression indicated its potential in mitigating heat stress-induced damage via multiple pathways. HSPs are pivotal in modulating the inflammatory response under stress conditions. Study indicated that HSPs induction and cytokine production in immune cells could be mediated through the NF-κB signaling pathway [57].

HSPs were capable of inducing immune cells to release pro-inflammatory cytokines, such as TNF-α, IL-1β, and IL-6 [58,59]. The inflammatory status across groups was further assessed by measuring key cytokine expression levels. As shown in Figure 5C–E, heat stress significantly upregulated the mRNA expression of pro-inflammatory factors Interleukin-6 (IL-6), Interleukin-1β (IL-1β), and Tumor Necrosis Factor α (TNF-α) in the HS group compared to the NCD group (*p* < 0.05). MBS intervention significantly counteracted this effect, with both HS-NMBS and HS-RMBS groups showing marked downregulation of all three pro-inflammatory cytokines relative to the HS group (*p* < 0.05). Noticeably, the suppression of IL-1β was significantly stronger in the HS-NMBS group than in the HS-RMBS group (*p* < 0.05). For anti-inflammatory response (Figure 5F), the HS-NMBS group exhibited significant upregulation of IL-10 expression compared to the HS group (*p* < 0.05), reaching a level comparable to that of the NCD group. A significant difference in IL-10 expression was also observed between the HS-NMBS and HS-RMBS groups (*p* < 0.05). Lu, Hu, Liu, Yu, Hu, Jiang, Liu, Li, He, Yang and Liang [25] found a significant increase and decrease in the gene expression levels of TNF-α and IL-10 in heat-stressed mice, respectively. Similar results were found in heat stress-induced inflammation in rats [60] and chickens [61]. Our findings suggested that MBS supplementation effectively mitigated heat stress-induced inflammatory dysregulation by restoring the balance between pro- inflammatory and anti-inflammatory mediators. The significant suppression of IL-1β and the unique ability to upregulate IL-10 observed with NMBS might be attributable to the retention of vitexin and isovitexin, which have been shown to exert anti-inflammatory activities through multiple pathways [35]. In addition, studies have shown that chlorogenic acid and *p*-coumaric acid could alleviate inflammatory responses by inhibiting the activation of the NF-κB and MAPK signaling pathways [62,63]. The significantly reduced levels of these compounds in RMBS might further compromise its anti-inflammatory potential. Publicly available stress-response transcriptomic data (Atlas of Stress Response Activity) [64] have demonstrated that HSP/HSPA family genes were commonly upregulated under diverse stressors, including heat stress and oxidative stress, reflecting their conserved functions in maintaining protein homeostasis. Likewise, inflammation-related genes (IL1-β, IL-6, TNF-α, and I-L10), have also been shown to exhibit stress-induced expression remodeling across multiple cell-line models. In this context, the coordinated changes in HSP70, PPAR-γ, and these cytokines observed in our study suggested that heat stress activated a comprehensive stress-response program involving both proteostatic disturbance and inflammatory dysregulation.

Collectively, the simultaneous modulation of HSP70, PPAR-γ, and multiple cytokines by MBS suggested that its protective effects involved multi-target modulation of stress-responsive pathways, rather than a single antioxidative mechanism. Furthermore, NMBS conferred superior protection compared to RMBS, potentially due to its higher content of bioactive phenolic constituents. However, given that these findings were based exclusively on mRNA expression data, further validation of the underlying mechanisms at the protein level via Western blotting was warranted.

### 3.9. The Effect of NMBS and RMBS on the Gut Microbiota

#### 3.9.1. The Diversity and Richness of Gut Microbiota

The results of 16S rRNA sequencing analysis showed that a total of 1,288,302 high-quality sequences were obtained from the 24 samples, with an average of 53,679 sequences. The rarefaction curves and Shannon–Wiener curves both reached stable asymptotes with increasing sequencing depth, confirming adequate sampling effort and data saturation (Figure S1A,B).

**Figure 6:**
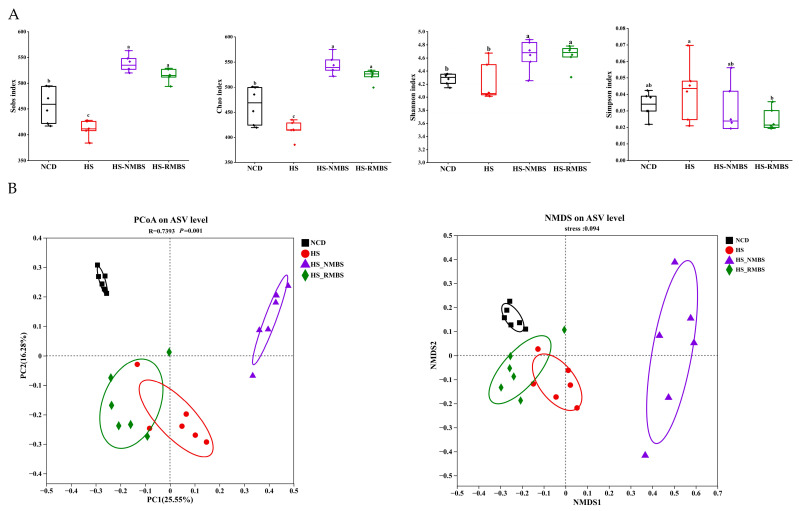
Effects of NMBS and RMBS on alpha and beta diversity of gut microbiota. (**A**) Alpha diversity. (**B**) Beta diversity assessed by principal coordinate analysis (PCoA) and non-metric multidimensional scaling (NMDS) score plots. Different letters indicated statistically significant differences between groups (*p* < 0.05), whereas values sharing the same letter were not significantly different (*p* ≥ 0.05).

Microbial α-diversity analysis (Figure 6A) revealed that the HS group exhibited significantly lower Sobs and Chao indices compared to the NCD group (*p* < 0.05). However, no significant differences were observed in the Shannon and Simpson indices between the HS and NCD groups. These results collectively indicated that heat stress significantly compromised the species richness (ACE and Chao1 indices) of the gut microbiota in mice, while exerting no marked effect on overall community diversity (Shannon and Simpson indices). However, MBS intervention effectively counteracted these changes, with both HS-NMBS and HS-RMBS groups showing significantly elevated Sobs, Chao, and Shannon indices as compared with the HS group (*p* < 0.05). Furthermore, no significant differences were observed between the HS-NMBS and HS-RMBS groups. In addition, β-diversity was assessed at the ASV level using principal coordinate analysis (PCoA) and non-metric multidimensional scaling (NMDS) (Figure 6B). The NCD group formed a tight cluster, while the HS and HS-RMBS groups showed partial overlap. In contrast, the HS-NMBS group was clearly separated from all other groups. The ANOSIM test results (*p* = 0.001, *R* = 0.7393) confirmed this observation. NMDS also corroborated these findings, showing the NCD group clustered in the upper left quadrant, the HS and HS-RMBS groups overlapping in the central-lower region, and the HS-NMBS group distinctly separated from other groups.

The observed reduction in α-diversity indices under heat stress aligned with previous findings by Wang, et al. [65] demonstrating the detrimental impact of heat stress on murine gut microbiota. Our results highlighted the efficacy of MBS in mitigating heat stress-induced microbial dysbiosis. The β-diversity patterns provided compelling evidence that heat stress substantially altered the gut microbiota composition. Although the HS and HS-RMBS groups exhibited partial overlap, the HS-NMBS group was completely clustered away from the HS group. Collectively, these findings demonstrated that heat stress markedly perturbed the gut microbiota, resulting in a community structure distinct from that of the NCD. MBS intervention effectively attenuated these heat stress-induced alterations, underscoring its pivotal role in modulating the structure of gut microbiota.

#### 3.9.2. The Gut Microbiota Composition

**Figure 7:**
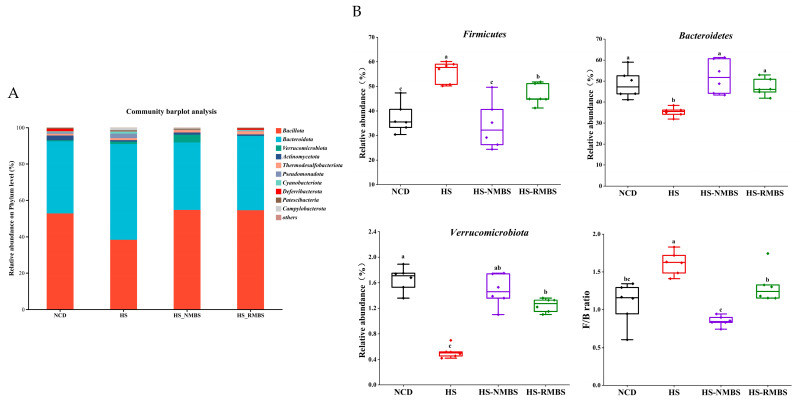
Effects of NMBS and RMBS on gut microbiota composition at the phylum level in mice. (**A**) Relative abundance of the major bacterial at the phylum level. (**B**) Relative abundance of *Firmicutes*, *Bacteroidetes*, *Verrucomicrobiota*, and the *Firmicutes*/*Bacteroidetes* (F/B) ratio. Different letters indicated statistically significant differences between groups (*p* < 0.05), whereas values sharing the same letter were not significantly different (*p* ≥ 0.05).

To determine the changes in the composition of gut microbial, the relative abundance was compared at the phylum and genus level. At the phylum level, the proportion of *Firmicutes*, *Bacteroidetes*, and *Verrucomicrobiota* reached more than 90% and was significantly different between the groups (Figure 7A). Compared with the NCD group, HS significantly reduced the relative abundance of *Bacteroidetes* and *Verrucomicrobia*, but significantly increased the relative abundance of *Firmicutes* (Figure 7B, *p* < 0.05). However, NMBS and RMBS supplementation significantly reversed these trends. Noticeably, HS significantly elevated the ratio of *Firmicutes*/*Bacteroidetes* (F/B). MBS intervention markedly reduced the F/B ratio, with the HS-NMBS group demonstrating a significantly greater reduction than that of the HS-RMBS group (*p* < 0.05).

**Figure 8:**
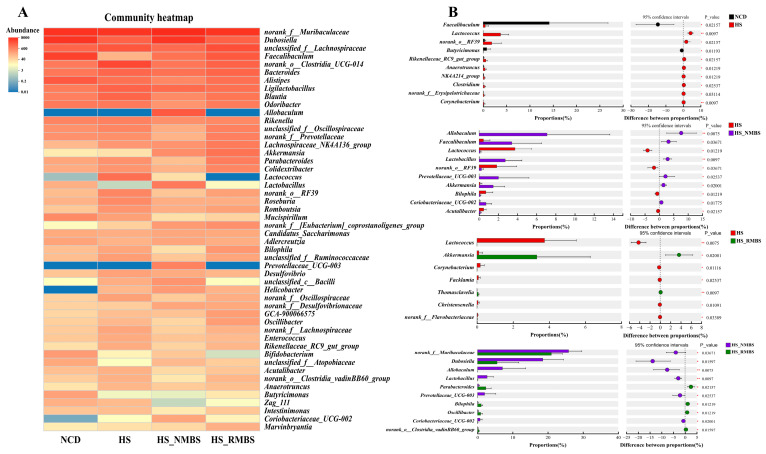
Effects of NMBS and RMBS on gut microbiota composition at the genus levels in mice. (**A**) Relative abundance at the genus level (top 50 most abundant genera). (**B**) Wilcoxon rank-sum test was performed to assess differences in the relative abundance of key genera between the following two groups: NCD vs. HS, HS vs. HS-NMBS, HS vs. HS-RMBS, and HS-NMBS vs. HS-RMBS. * 0.01 < *p* ≤ 0.05, ** 0.001 < *p* ≤ 0.01.

The top 50 most abundant genera at the genus level are shown in Figure 8A. To further explore bacteria that may confer protection against heat stress, we analyzed significant differences in the relative abundance of key genera (Figure 8B). Specifically, compared with the NCD group, the HS group exhibited significantly increased relative abundances of *Lactococcus*, *norank_o__RF39*, *Clostridium*, while the relative abundances of *Faecalibaculum*, *Butyricimonas* were significantly decreased. The interventions of NMBS and RMBS had some commonalities in the modulation of certain genera, both of which significantly increased the relative abundance of the *Akkermansia* and reduced the relative abundance of *Lactococcus* compared to the HS group. However, compared to the HS-RMBS group, the HS-NMBS group exhibited significantly higher relative abundances of *norank_f__Muribaculaceae*, *Dubosiella*, *Allobaculum*, and *Lactobacillus*. In addition, NMBS and RMBS were each targeted to promote or inhibit the growth of certain specific genera. For example, the NMBS intervention promoted the growth of *Allobaculum*, *Faecalibaculum*, and *Lactobacillus*, and inhibited the growth of *Bilophila*, *norank_o__RF39*, and *Acutalibacter*. In contrast, the RMBS intervention increased the relative abundance of *Thomasclavelia* and decreased the relative abundance of *Corynebacterium* and *Facklamia*.

#### 3.9.3. The Effect of NMBS and RMBS on Key Phylotypes of the Gut Microbiota

**Figure 9:**
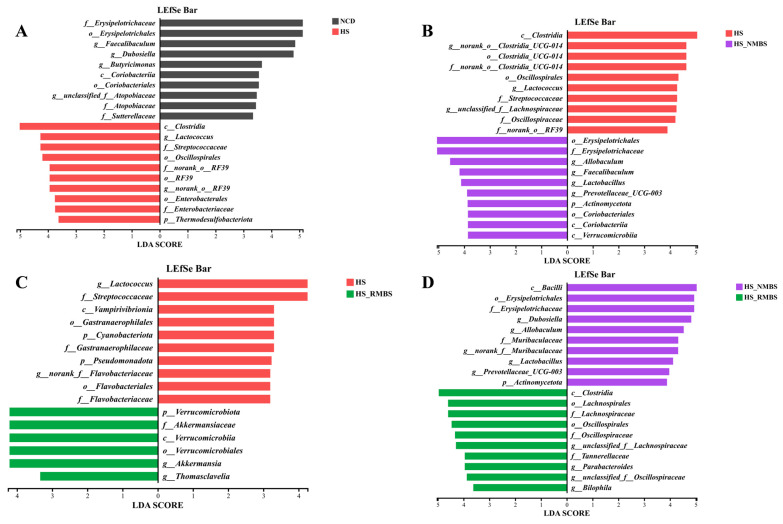
LDA scores obtained from LEfSe analyses, LDA score > 2.0. (**A**) NCD vs. HS. (**B**) HS vs. HS-NMBS. (**C**) HS vs. HS-RMBS. (**D**) HS-NMBS vs. HS-RMB.

LEfSe was used to identify dominant bacteria from phylum to genus via linear discriminant analysis (LDA). Integrating the cladogram with LDA scores (threshold > 2) revealed significant differentially abundant bacteria (Figure 9A–D). These identified specific bacterial taxa were enriched by the MBS supplementation. The results indicated that the key phylotypes of the gut microbiota in response to NMBS supplementation were the bacterial genera *Allobaculum*, *Faecalibaculum*, and *Lactobacillus*. In contrast, the key responsive phylotypes to RMBS supplementation were mainly *Akkermansia* and *Thomasclavelia*. Furthermore, comparative analysis between the HS-NMBS and HS-RMBS groups revealed that NMBS supplementation significantly enriched *Lactobacillus*, *Dubosiella*, and *norank_f_Muribaculaceae*. Collectively, these results demonstrated that gut microbiota dysbiosis induced by heat stress was significantly modulated by MBS supplementation.

Studies have shown that heat stress exacerbated and worsened intestinal diseases, and one of its mechanisms of action may be the alteration of gut microbiota composition [66,67]. Furthermore, a previous study has demonstrated that an elevated F/B ratio could trigger inflammatory responses [68]. The study of Liu, et al. [69] has demonstrated that heat stress exposure led to an elevated relative abundance of *Firmicutes*, alongside reduced abundances of *Bacteroidetes*, which was consistent with our findings. In this study, both NMBS and RMBS supplementation significantly reversed the heat stress-induced increase in the relative abundances of *Firmicutes*, while concurrently reducing the relative abundance of *Bacteroidetes*, thereby lowering the F/B ratio. These findings substantiated that MBS intervention effectively counteracted heat stress-induced gut dysbiosis at the overall microbial structural level.

Furthermore, analysis at the genus level can help elucidate the relationships between specific bacterial taxa and their biological benefits. Research has shown that heat stress altered the composition of the gut microbiota in livestock and poultry, leading to an increase in pathogenic bacteria and a decrease in beneficial microbes [70]. *Akkermansia* and *Lactobacillus* are recognized as key beneficial genera, demonstrating positive impacts on human health [71,72]. *Akkermansia* is a mucin-degrading bacterium, which has been shown to alleviate heat stress-induced intestinal barrier dysfunction [73]. In addition, *Lactobacillus* can also ameliorate heat stress-induced intestinal dysfunction by enhancing barrier function and suppressing inflammatory responses [74]. A previous investigation demonstrated that heat stress significantly reduced the abundances of *Lactobacillus*, *Allobaculum*, *Akkermansia*, *Bacteroides*, and *Faecalibaculum* [65], which was consistent with our findings. In this study, both NMBS and RMBS interventions significantly increased the relative abundance of *Akkermansia*. Thus, the benefits of MBS can be partially attributed to its modulation of this gut microbiota. Furthermore, *Faecalibaculum* levels were significantly elevated following NMBS supplementation. Additionally, MBS supplementation suppressed the proliferation of harmful bacteria such as *Bilophila* and *norank_f_Lachnospiraceae*. Studies have indicated that heat stress led to an increased relative abundance of *Bilophila* and *norank_f_Lachnospiraceae* in the host [75,76]. In this study, heat stress also led to a significant increase in the relative abundance of *Lactococcus*, while subsequent MBS intervention significantly reduced it. However, a previous study showed discrepant results. The study found that *Bacillus licheniformis* intervention significantly increased the relative abundance of *Lactococcus* in heat-stressed rats, thereby enhancing gut barrier function [7]. The duration of heat stress intervention may be the primary reason for discrepancies in the results. The LEfSe analysis further revealed that the HS-NMBS group was characterized by significant enrichments in *Lactobacillus*, *Dubosiella*, and *norank_f_Muribaculaceae*. Among them, *Dubosiella* has been demonstrated to elevate SOD levels, thereby mitigating damage induced by oxidative stress [77]. Meanwhile, *norank_f_Muribaculaceae* showed a negative correlation with inflammatory cytokine levels [78]. Accumulating evidence indicated that phenolic compounds and their metabolites of mung bean could modulate gut microbial community structure by exerting selective antimicrobial effects against pathogenic bacteria while concurrently promoting the growth of beneficial commensals [79]. For instance, vitexin has been reported to ameliorate gut dysbiosis in DSS-induced colitis mice by increasing the abundance of beneficial bacteria (e.g., *Alistipes*) while reducing that of harmful bacteria, including *norank_f_Lachnospiraceae* and *Candidatus_Saccharimonas* [80]. Furthermore, the ethanol extract of mung beans, whose major bioactive constituents were vitexin, isovitexin, and catechin, could alleviate alcohol-induced hepatointestinal injury through the specific in vivo enrichment of *Lactobacillus johnsonii* [81]. Consequently, the marked reduction in phenolic compounds, especially vitexin and isovitexin, in RMBS may compromise its capacity to support these beneficial bacteria. Many probiotic properties were species-specific. To better interpret the genus-level changes observed in this study, we summarized previously reported properties of representative species within the genera of interest (Table 3). The documented anti-inflammatory, antioxidant, and barrier-protective functions of these species were largely consistent with our observed genus-level shifts, providing a plausible mechanistic rationale for the protective effects of MBS against heat stress. However, whether the microbiota modulation induced by MBS played a dominant role in mediating the alleviation of oxidative stress, inflammatory responses, and tissue protection warranted further investigation through validation experiments, such as fecal microbiota transplantation or germ-free animal models.

## 4. Conclusions

This study suggested that both NMBS and RMBS alleviated heat stress-induced physiological damage in mice. However, the red discoloration of MBS during storage was accompanied by a significant loss of phenolic compounds, particularly vitexin and isovitexin, which might contribute to a marked reduction in its efficacy against heat stress. Consequently, NMBS provided more pronounced protection by more effectively downregulating the expression of heat stress-related genes (PPAR-γ) and pro-inflammatory cytokines (e.g., IL-1β), while more potently upregulating the expression of anti-inflammatory cytokine IL-10. Furthermore, NMBS intervention was more effective in restoring gut microbiota diversity, reducing the F/B ratio towards normal levels, and specifically enriching beneficial bacteria such as *Lactobacillus*, *Dubosiella*, and *norank_f_Muribaculaceae*. These findings highlighted the importance of preserving the fresh state of mung bean soup to maximize its potential as a natural dietary intervention against heat stress. Future studies should focus on optimizing processing and storage conditions to minimize phenolic degradation. Notably, future research should validate the findings of transcriptomic studies at the protein level and evaluate tight junctions (occludin, claudin-1, and ZO-1) and intestinal permeability to further elucidate barrier protection mechanisms. Furthermore, only male mice were used in this study, and whether the observed protective effects of MBS extended to female subjects remained to be determined. Future studies incorporating both sexes are warranted to verify these findings and to explore potential sex-specific mechanisms. In particular, further investigation is warranted to comprehensively characterize the degradation products and elucidate the underlying transformation mechanisms, thereby providing a molecular basis for quality control and efficacy preservation of mung bean soup.

## Figures and Tables

**Table 1:** The phenolic compounds of NMBS and RMBS (mg/100 g).

Categorization	Phenolic Compounds	NMBS	RMBS
Phenolic acids	Chlorogenic acid	1.07 ± 0.05 ^a^	0.54 ± 0.06 ^b^
	*p*-Coumaric acid	0.44 ± 0.14 ^a^	0.04 ± 0.07 ^b^
	Vanillic acid	0.54 ± 0.04 ^a^	0.12 ± 0.03 ^b^
	Gallic acid	0.33 ± 0.02 ^a^	0.16 ± 0.01 ^b^
	Benzoic acid	0.42 ± 0.01 ^a^	0.07 ± 0.01 ^b^
	Ferulic acid	0.28 ± 0.01 ^a^	0.12 ± 0.01 ^b^
Catechins	Catechin	1.79 ± 0.02 ^a^	0.44 ± 0.01 ^b^
	Epicatechin	0.65 ± 0.02 ^a^	0.47 ± 0.03 ^b^
Flavonoids	Quercetin	0.31 ± 0.08 ^b^	0.37 ± 0.11 ^a^
	Naringin	1.66 ± 0.11 ^a^	1.04 ± 0.01 ^b^
	Diosmetin	0.36 ± 0.08 ^b^	0.41 ± 0.05 ^a^
	Naringenin	0.10 ± 0.01 ^b^	0.11 ± 0.01 ^b^
	Vitexin	37.71 ± 1.32 ^a^	15.23 ± 0.78 ^b^
	Isovitexin	47.85 ± 1.45 ^a^	17.08 ± 1.26 ^b^
	Tangeretin	5.01 ± 0.01 ^a^	4.99 ± 0.01 ^a^
	Vanillin	0.08 ± 0.01 ^a^	0.03 ± 0.00 ^b^
	Rutin	2.91 ± 0.12 ^a^	1.38 ± 0.07 ^b^

Different letters in the same row indicated statistically significant differences between groups (*p* < 0.05), whereas values sharing the same letter were not significantly different (*p* ≥ 0.05).

**Table 2:** Effects of NMBS and RMBS on organ indices in mice.

Group	Liver Index (%)	Kidney Index (%)	Thymus Index (%)	Spleen Index (%)
NCD	5.13 ± 0.15 ^a^	1.27 ± 0.01 ^a^	0.17 ± 0.00 ^a^	0.33 ± 0.00 ^a^
HS	3.99 ± 0.09 ^d^	1.17 ± 0.04 ^b^	0.05 ± 0.01^c^	0.26 ± 0.01 ^b^
HS-NMBS	4.58 ± 0.07 ^b^	1.19 ± 0.03 ^b^	0.10 ± 0.01 ^b^	0.32 ± 0.02 ^a^
HS-RMBS	4.53 ± 0.15 ^bc^	1.24 ± 0.03 ^bc^	0.11 ± 0.01 ^b^	0.32 ± 0.01 ^a^

The different letters in the same row meant significant difference among groups (*p* < 0.05).

**Table 3:** Previously reported host-beneficial properties of key bacterial genera.

Genus	Representative Specie	Reported Properties	References
*Akkermansia*	*A. muciniphila*	Protection against heat-induced intestinal barrier dysfunction via HSP27 phosphorylation and upregulation of tight junction proteins (Occludin, ZO-1)	[73]
*Lactobacillus*	*L. plantarum* 4-2	Alleviation of heat stress-induced oxidative stress and ileal damage via Keap1-Nrf2; modulation of pro-/anti-inflammatory cytokine balance (increased *Il-10*/*Tgf-β1*, decreased *Ifn-γ*/*Il-6*)	[82]
*Dubosiella*	*D. newyorkensis*	Reduction in oxidative stress (decreased MDA, increased SOD) and improvement of vascular endothelial function	[77]
*Faecalibaculum*	*F. rodentium*	Prevention of intestinal inflammation via gut bacteria-mediated histidine biosynthesis.	[83]
*Allobaculum*	*A. stercoricanis*	Prevention of NAFLD progression through inhibition of hepatic fat accumulation under metabolic stress.	[84]
*norank_f_Muribaculaceae*	N.F.	Production of short-chain fatty acids, regulation of intestinal barrier function and immune responses, and considered a promising “next generation probiotic”	[85]

N.F.: not found.

## Data Availability

The raw 16S rRNA sequencing data generated in this study have been deposited in the NCBI Sequence Read Archive (SRA) under BioProject accession number PRJNA1491072. All other data presented in this study are available in this paper.

## Supplementary Materials

The following supporting information can be downloaded at https://www.mdpi.com/article/10.3390/nu18142353/s1, Figure S1: Alpha diversity analysis of samples by Rarefaction analysis (A) and Shannon index (B). Each bar represents one sample; Table S1: Primer sequences; Excel files: Relative abundances of gut microbiota at the phylum and genus level based on ASVs.
