# Supplementary material for: A Comparison Between Normal and Reddened Mung Bean Soup: Beneficial Effects on the Regulation of Oxidative Stress, Inflammation, and Gut Microbiota in Mice with Heat Stress-Induced Damages
Source: Nutrients. 2026 Jul 17;18(14):2353. doi: 10.3390/nu18142353 (PMC13414598; doi:10.3390/nu18142353)
Supplement: Supplementary file 1 [file nutrients-18-02353-s001.zip › Supplementary Materials.pdf]

Table S1 Primer sequences

Gene	Forward sequence (5'-3')	Reverse sequence (5'-3')
GAPDH	GTTTCCTCGTCCCGTAG	AATCTCCACTTTGCCACT
HSP70	CTGCCGCTGAGAGTCGTTGAAG	GCACTAGCCAGGAGGGAGAACAG
PPARγ	GATTTCTCCAGCATTTC	ATCGCACTTTGGTATT
1L-1β	TCGCAGCAGCACATCAACAAGAG	TGCTCATGTCCTCATCCTGGAAGG
1L-10	AGAGAAGCATGGCCCAGAAATCAAG	CTTCACCTGCTCCACTGCCTTG
TNF-α	CCCTCACACTCACAAACCACC	CTTTGAGATCCATGCCGTTG

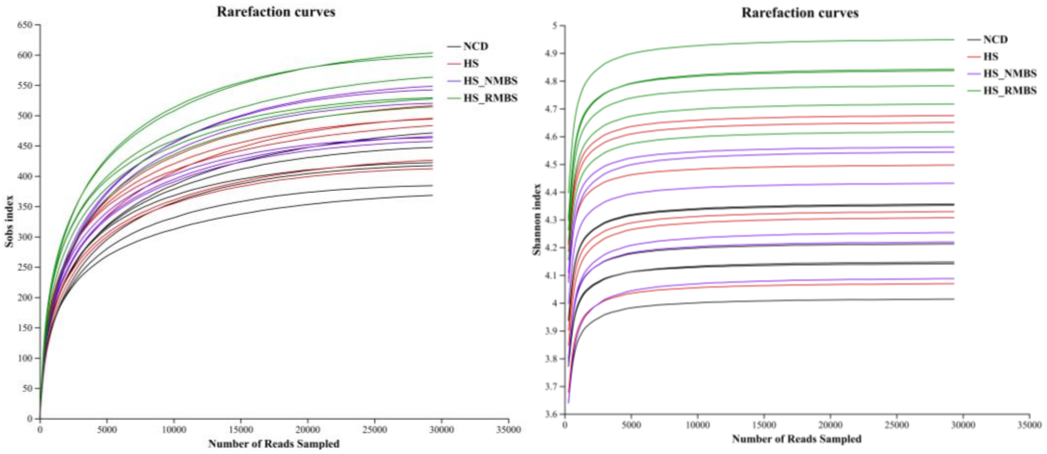

Figure S1 Alpha diversity analysis of samples by Rarefaction analysis (A) and Shannon index (B). Each bar represents one sample.
